# miR-30e Blocks Autophagy and Acts Synergistically with Proanthocyanidin for Inhibition of AVEN and BIRC6 to Increase Apoptosis in Glioblastoma Stem Cells and Glioblastoma SNB19 Cells

**DOI:** 10.1371/journal.pone.0158537

**Published:** 2016-07-07

**Authors:** Mrinmay Chakrabarti, Daniel J. Klionsky, Swapan K. Ray

**Affiliations:** 1 Department of Pathology, Microbiology, and Immunology, University of South Carolina School of Medicine, Columbia, South Carolina, United States of America; 2 Life Sciences Institute, University of Michigan, Ann Arbor, Michigan, United States of America; Swedish Neuroscience Institute, UNITED STATES

## Abstract

Glioblastoma is the most common and malignant brain tumor in humans. It is a heterogeneous tumor harboring glioblastoma stem cells (GSC) and other glioblastoma cells that survive and sustain tumor growth in a hypoxic environment via induction of autophagy and resistance to apoptosis. So, a therapeutic strategy to inhibit autophagy and promote apoptosis could greatly help control growth of glioblastoma. We created hypoxia using sodium sulfite (SS) for induction of substantiated autophagy in human GSC and glioblastoma SNB19 cells. Induction of autophagy was confirmed by acridine orange (AO) staining and significant increase in Beclin-1 in autophagic cells. microRNA database (miRDB) search suggested that miR-30e could suppress the autophagy marker Beclin-1 and also inhibit the caspase activation inhibitors (AVEN and BIRC6). Pro-apoptotic effect of proanthocyanidin (PAC) has not yet been explored in glioblastoma cells. Combination of 50 nM miR-30e and 150 μM PAC acted synergistically for inhibition of viability in both cells. This combination therapy most effectively altered expression of molecules for inhibition of autophagy and induced extrinsic and intrinsic pathways of apoptosis through suppression of AVEN and BIRC6. Collectively, combination of miR-30e and PAC is a promising therapeutic strategy to inhibit autophagy and increase apoptosis in GSC and SNB19 cells.

## Introduction

Glioblastoma is a perpetually fatal central nervous system tumor, which generally occurs in the cerebral hemispheres and brain stem. Glioblastoma is composed heterogeneous tumor cells that can invade surrounding normal brain tissues and spread anywhere in the brain and spinal cord. In spite of surgery, radiation, and chemotherapy, patients with aggressive glioblastoma have shown a median survival of about 14.6 months only [1]. Thus, there is an urgent need to understand the molecular and cellular mechanisms of pathogenesis in glioblastoma and invent new therapeutic strategies to improve patient outcome.

Autophagy, which is an acclaimed cell survival strategy in solid tumors like glioblastoma, plays a crucial role in homeostatic removal with degradation and recycling of damaged and mis-folded proteins and organelles [2–4]. Recent investigations suggest that autophagy can be an important catabolic mechanism in solid tumors that can help in utilizing nutrients and providing building blocks for growth of tumor cells during starvation and hypoxia and thus, autophagy contributes to overall survival of the tumor cells [5,6]. As a result of uncontrolled growth of tumor cells, oxygen depletion or hypoxic microenvironment could contribute to survival strategy by inducing autophagy [7]. Many earlier investigations have described that autophagy can play a dual role in cell survival as well as in cell death; however, crosstalk and interplay between autophagy and apoptosis appear to be complex and also controversial [4,8].

MicroRNAs (miRs) play a crucial role in cellular differentiation and proliferation, and miRs have been widely investigated in variety of cancers including glioblastoma. Thus, modulation of expression of specific miRs in highly tumorigenic and self-renewing glioblastoma stem cells (GSC), which express the cell surface marker CD133^+^ [9,10], can offer a potential therapeutic approach to improving patient outcome. A recent study showed that miR-124 and miR-137 could induce neuronal differentiation in mouse oligodendroglioma stem cells (mOSC) and GSC as well and inhibit proliferation in other glioblastoma cell lines [11]. Thus, introduction of expression of specific miRs could be a useful therapeutic strategy for treatment of human glioblastoma.

Plant-derived polyphenols offer effective chemotherapeutic strategies for different types of cancers including glioblastoma. Many epidemiological studies indicated the concept that consumption of dietary polyphenols could reduce the risk of many cancers [12,13]. Proanthocyanidin (PAC), which is a bioactive phytochemical isolated from grape seed, has shown anti-carcinogenic activity in several animal tumor models [14–16]. Recent investigations showed anti-inflammatory, anti-oxidant, and anti-metastatic properties of PAC in both *in vitro* and *in vivo* models [14–18]. PAC could inhibit cell proliferation and induce apoptosis in various cell lines derived from different types of cancers including breast, colon, and prostate cancers [16–19]. A recent study demonstrated remarkable inhibition in cell viability in an esophageal adenocarcinoma cell line due to cell cycle arrest and induction of apoptosis following exposure to PAC [20]. However, there are only a few studies that show the anti-tumor potentials of PAC in human glioblastoma cells. Notably, oligomer procyanidins from grape seeds promoted apoptotic cell death in human glioblastoma U87 cells [21–22].

In our current study, inhibition of autophagy and induction of apoptosis by combination of a genetic material (miR) and a less toxic plant-derived pharmacological agent were explored for controlling the growth of human GSC and glioblastoma SNB19 cells in cultures. It is well known that GSC may remain resistant to radiotherapy and chemotherapy resulting in tumor recurrence. In this work, we targeted the highly resistant GSC and also SNB19 (PTEN mutant) cells under hypoxia condition using the hypoxia mimetic compound sodium sulfite (SS) to demonstrate the molecular mechanisms and potential of our novel combination of miR-30e and PAC for inhibition of autophagy and induction of apoptosis. No work has yet been reported to understand the mode of action of these agents as a combination therapy in human GSC and other glioblastoma cells. Our results on the GSC and SNB19 cells indicated that the combination of miR-30e and PAC could be a novel therapeutic strategy that might be used for controlling of the growth of different human glioblastoma cells.

## Materials and Methods

### Human glioblastoma cell lines and culture conditions

Human glioblastoma stem cells (GSC) and glioblastoma SNB19 cells were purchased from Celprogen (San Pedro, CA, USA) and American Type Culture Collection (ATCC, Manassas, VA, USA), respectively. GSC was grown in stem cell media (Mediatech, Manassas, VA, USA) while SNB19 was propagated in DMEM (Mediatech, Manassas, VA, USA) supplemented with 10% fetal bovine serum (FBS) (Atlanta Biologicals, Lawrenceville, GA, USA), 1% penicillin, and 1% streptomycin (GIBCO, Grand Island, NY, USA) in a fully-humidified incubator (Napco series 8000 WJ, Tharmo scientific) containing 5% CO_2_ at 37°C. SS and PAC were procured from Sigma Chemical (St. Louis, MO, USA). PAC was dissolved in dimethyl sulfoxide (DMSO) to make stock solution and aliquots of the stock solution were stored at -20°C in dark until ready to use. Final concentrations of DMSO in cell cultures were maintained at less than 0.01% that did not affect cell growth or death.

### Acridine orange (AO) staining to observe and quantify acidic vesicular organelles (AVO) in autophagic cells

Formation of AVO is a characteristic feature of autophagic cells [3]. We performed AO staining to detect and quantify AVO after treatment with different doses of SS. Briefly, overnight grown cells were exposed to 0.1, 0.5, 1.0, and 2.0 mM SS for 24 h in slide cultures and then incubated with AO (1 μg/ml) for 15 min for staining. Another set of SS unexposed cells were used as control (CTL). After washing thrice with 1x phosphate-buffered saline (PBS, pH 7.4), all slides containing the AO stained cells were then observed under the fluorescence microscope to detect the formation of AVO. We also performed flow cytometric analysis (Beckman Coulter, Fullerton, CA, USA) after AO staining, as described above, of both GSC and SNB19 cells. In flow cytometric analysis of the AO stained cells, cytoplasm and nucleolus in non-autophagic cells showed green fluorescence (500–550 nm, FL-1 channel) whereas AVO in autophagic cells (quadrant A1) showed bright red fluorescence (650 nm, FL-3 channel). Red fluorescence intensity is directly proportional to the number of AVO in autophagic cells. Therefore, on the basis of red fluorescence intensity, formation of AVO in autophagic cells can be determined.

### Transfection of cells with miR-30e and treatment with PAC

Both GSC and SNB19 cells were allowed to grow till 80–90% confluency, treated with 2 mM SS for 24 h, and then transfected with 25, 50, or 100 nM miR-30e (Darmacon, Chicago, IL, USA) following the manufacturer’s protocol. Transfection was performed using Lipofectamine 2000 reagent (Invitrogen, Grand Island, NY, USA) following the manufacturer’s instruction. Briefly, SS pre-treated cells were seeded in a 24-well plate in 500 μl of growth medium (without antibiotics) and allowed to grow till 80–90% confluency. Then, both miR-30e and Lipofectamine 2000 reagent were diluted in 50 μl of serum-free Opti-MEM (Invitrogen, Grand Island, NY, USA) medium separately and incubated for 5 min. After incubation, the transfection mixtures were gently spread in each well. All cells were incubated at 37°C for 12 h and then treated with 50, 100, or 150 μM PAC for another 24 h and cell viability assay was performed.

### Evaluation of the residual cell viability by 3-(4,5-dimethylthiazolyl-2)-2,5-diphenyl-tetrazoliumbromide (MTT) assay

The MTT assay was performed to determine residual cell viability after transfection of cells with miR-30e and treatment with PAC as described above. The SS pre-treated GSC and SNB19 cells were either transfected with miR-30e (25, 50, or 100 nM) for 12 h or treated with PAC (50, 100, or 150 μM) for 24 h or combination of both agents. At the end of all treatments and incubation, old media were removed and cells were exposed to the 0.2 mg/ml MTT reagent (50 μl/well) for 3 h at 37°C followed by addition of isopropanol (100 μl) to dissolve the formazan crystals. The optical density (OD) was measured at 570 nm in a microplate reader (BioTek, Winooski, VT, USA). Cell viability data obtained from the MTT assay were then subjected to analysis by CompuSyn software (ComboSyn, Paramus, NJ, USA) to determine combination index (CI) values. Conventionally, CI > 1 indicates antagonism, CI = 1 indicates additive effect, and CI < 1 indicates synergism of the agents in combination.

### Evaluation of apoptosis by *in situ* Wright staining and also Annexin V-fluorescein isothiocyanate (FITC)/propidium iodide (PI) binding assay

Following miR-30e transfection or/and PAC treatment, both GSC and SNB19 cells were centrifuged and then washed with PBS, pH 7.4. Then, cells were fixed in 95% (v/v) ethanol, air-dried, and subjected to *in situ* Wright staining using a kit (Fisher Scientific, Kalamazoo, MI, USA) according to the manufacturer’s instructions. The morphological changes of the cells were examined under the Olympus BX53 microscope (Olympus America, Center Valley, PA, USA) and digitally photographed. The morphological features of apoptotic cells included cell shrinkage, chromatin condensation, and membrane bound apoptotic bodies. About 300 cells in each treatment were counted from six randomly selected fields and the percentage of apoptotic cells was calculated from three separate sets of experiments. Flow cytometric analysis of Annexin V-FITC/PI stained cells were performed to determine an early molecular event (externalization of membrane phospholipids) of apoptosis that occurred after single therapy or combination therapy. In flow cytometric analysis, cells that were Annexin V-FITC negative and PI positive were considered as mechanically injured (quadrant A1), cells that were both Annexin V-FITC and PI positive (quadrant A2) were considered as late necrotic, cells that were both Annexin V-FITC and PI negative (quadrant A3) were considered as normal, and cells that were Annexin V-FITC positive and PI negative were considered as early apoptotic (quadrant A4). Flow cytometry detected the Annexin V-FITC positive cells that experienced externalization of membrane phospholipids, an early biochemical feature of apoptosis. The Annexin V-FITC stained apoptotic cells were analyzed for statistical significance.

### RNA extraction and reverse transcription-polymerase chain reaction (RT-PCR)

Following miR-30e transfection or/and PAC treatment, as described above, RT-PCR was carried out to determine the levels of mRNA expression of Beclin-1, glyceraldehyde 3-phosphate dehydrogenase (GAPDH), AVEN, and BIRC6 in both GSC and SNB19 cells. The mRNA expression of GAPDH was used as an internal standard. Briefly, total RNA was extracted from 3×10^6^ GSC or SNB19 cells using TRIZOL (Invitrogen, Carlsbad, CA, USA) according to the manufacturer's protocol. Then, the isolated RNA (300 ng) was used for each set of primers for transcription and amplification using a single-step RT-PCR kit (Invitrogen, Carlsbad, CA, USA) on a PCR cycler (Eppendorf, Westbury, NY, USA) with following programming: cDNA synthesis at 50°C for 30 min, reverse transcriptase inactivation at 95°C for 10 min, 35 cycles of PCR amplification of transcripts (denaturation of templates at 95°C for 45 s, annealing of primers at 52°C for 30 s, and extension of primers at 72°C for 1 min), and final extension of primers at 72°C for 7 min. Then, the RT-PCR products were resolved on 1% agarose gels, stained with ethidium bromide (1 μg/ml), and visualized on a UV (303 nm) transilluminator and digitally photographed using the UVDI Compact Digimage System (Major Science, Saratoga, CA, USA).

### Western blotting to evaluate levels of specific molecules involved in autophagy and apoptosis

We performed sodium dodecyl sulfate-polyacrylamide gel electrophoresis (SDS-PAGE) and Western blotting to assess the levels of expression of specific signaling proteins involved in autophagy and apoptosis. Following different treatments, as described above, cells were homogenized in lysis buffer and protein concentrations were determined after staining the protein samples with Coomassie Plus protein reagent (Pierce Biotechnology, Rockford, IL, USA) using the modified Bradford method. Proteins were resolved by SDS-PAGE and the resolved proteins were transferred to the polyvinylidene fluoride (PVDF) membranes (Millipore, Billerica, MA, USA). After blocking in 5% non-fat milk, PVDF membranes were subsequently probed with specific primary IgG antibodies. We used rabbit primary antibodies against Beclin-1, LC3, TLR-4, mTOR, p62, AVEN, BIRC6, caspase-8, Bax, Bcl-2, calpain, caspase-9, caspase-3, SBDP, GAPDH, and β-actin (Santa Cruz Biotechnology, Santa Cruz, CA, USA) for our studies. For detection of primary IgG antibodies, we used horseradish peroxidase (HRP) conjugated secondary IgG antibodies (1:3000) followed by HRP-Immunostar chemiluminescence reagent (Bio-Rad Laboratories, Hercules, CA, USA). Then, Western blots were exposed to X-OMAT AR films (Eastman Kodak, Rochester, NY, USA) and autoradiograms were scanned on an EPSON Scanner using Photoshop software (Adobe Systems, Seattle, WA, USA) to determine alterations in expression of specific proteins. Expression of β-actin or GAPDH was used as a loading standard.

### Statistical analysis

The results from some of the experiments were analyzed for statistical significance using Minitab 16 statistical software (Minitab, State College, PA, USA). Data were expressed as mean ± standard error of mean (SEM) of separate experiments (*n* ≥ 3) and compared by one-way analysis of variance (ANOVA) followed by the Fisher's post-hoc test. Difference between a control (CTL) group and a treatment group was considered significant at *P* value less than 0.05.

## Results

### Sodium sulfite (SS) treatment for induction of autophagy in human GSC and SNB19 cells

**Fig 1:**
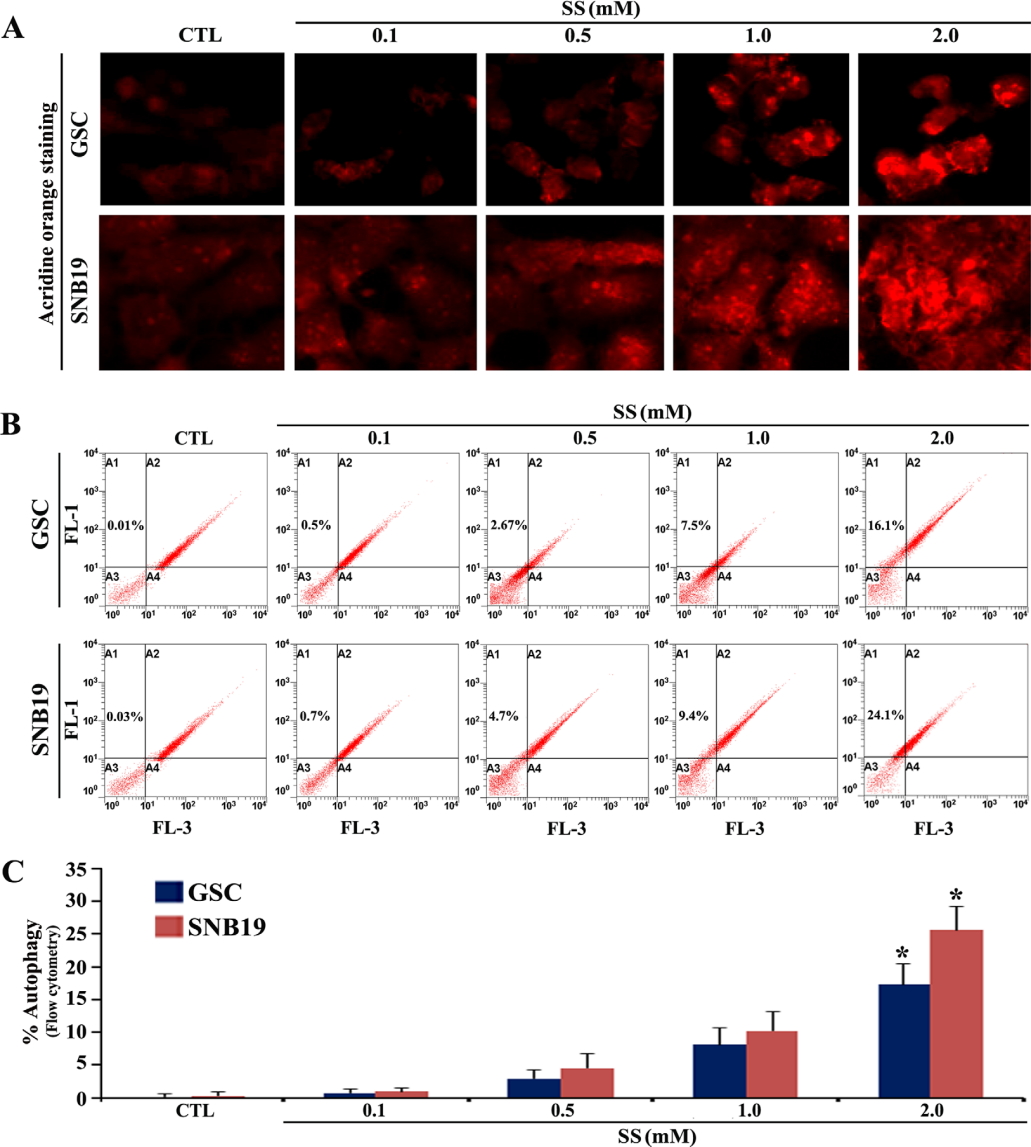
The AO staining to show induction of autophagy after exposure of GSC and SNB19 cells to SS. Overnight grown GSC and SNB19 cells were exposed to 0.1, 0.5, 1.0, and 2 mM SS for 24 h. Untreated cells were considered as control (CTL). (A) Staining of cells with AO followed by fluorescence microscopy for detection of AVO in autophagic cells. (B) Flow cytometric analysis of the AO stained cells from all treatment groups for detection and determination of AVO in autophagic cells. (C) Quantitative analysis of the autophagic cell populations shown in bar diagrams on the basis of flow cytometric data. Significant difference between CTL and any treatment was indicated by *P < 0.05.

We first investigated whether the hypoxia mimetic compound SS could induce autophagy (a typical cell survival strategy in solid tumor) in human GSC and SNB19 cells (Fig 1). Exposure of the cells to different doses (0.1–2 mM) of SS for 24 h followed by staining with acridine orange (AO) showed that 2 mM SS was very effective in inducing autophagy in both GSC and SNB19 cells (Fig 1A). Flow cytometric analysis of the AO stained cells showed the autophagic populations in both GSC and SNB19 cells (Fig 1B) and the results were also presented in the form of bar diagrams (Fig 1C). Approximately, 16% and 24% autophagic populations were observed in GSC and SNB19 cells, respectively, following exposure to 2 mM SS for 24 h.

### Increase in expression of Beclin-1 at mRNA and protein levels in autophagic cells

**Fig 2:**
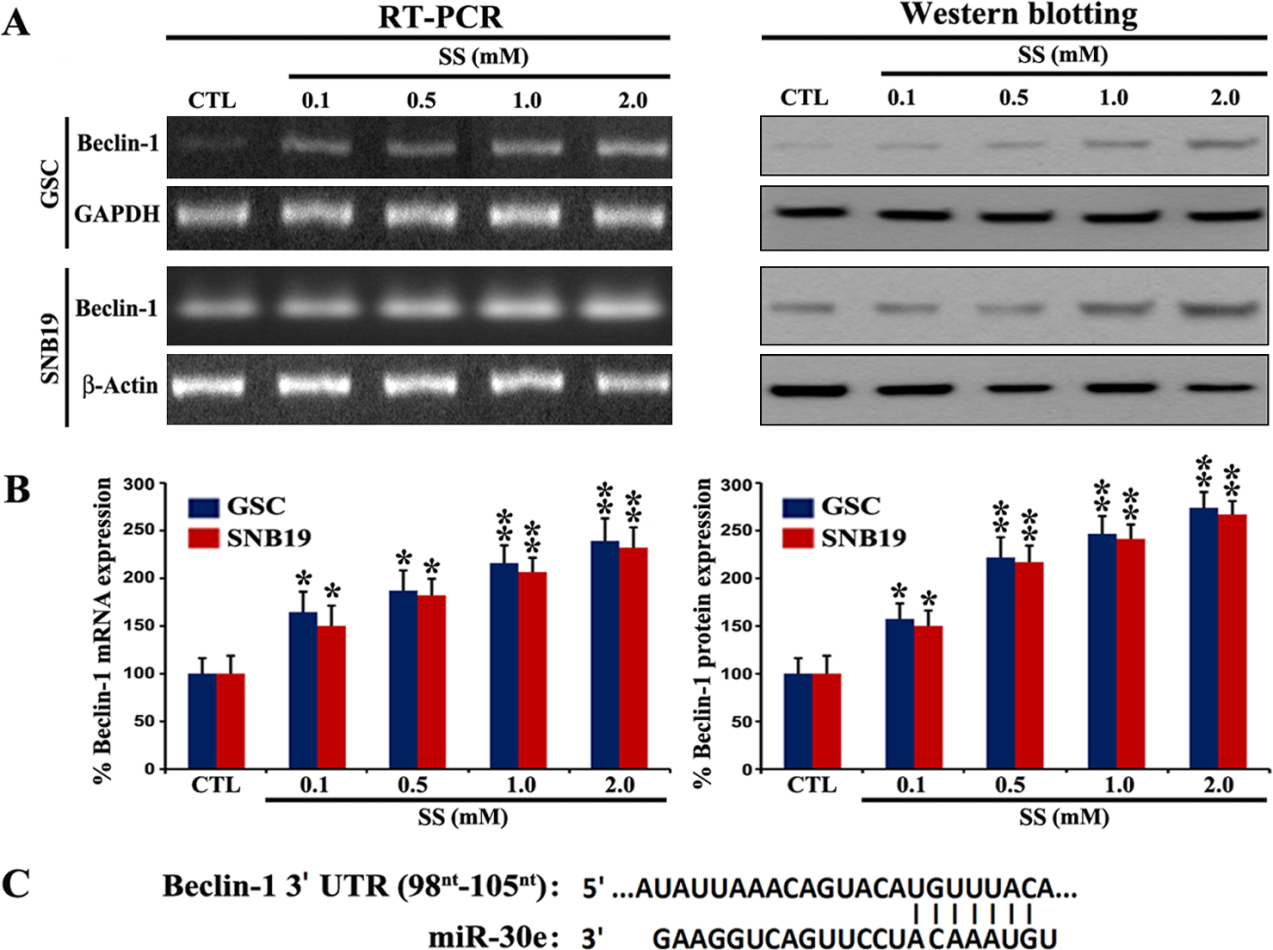
Changes in levels of expression of Beclin-1 in GSC and SNB19 cells corresponded with the miR-30e target prediction. (A) RT-PCR and Western blotting to determine relative expression of Beclin-1 mRNA and protein, respectively, in GSC and SNB19 cells after treatment with SS, as described in Fig 1 legend. (B) Quantitative analysis of Beclin-1 mRNA and protein from three independent experiments to present as bar diagrams. Significant difference between CTL and any treatment was indicated by *P < 0.05 and **P < 0.01. (C) Bioinformatics analysis showing Beclin-1 as a potential target for miR-30e.

Beclin-1, which is an early molecular marker of autophagy, is required for initiation of formation of the autophagosome in autophagy. Therefore, we tracked and confirmed the increase in expression of Becline-1, an early marker of autophagy, in GSC and SNB19 cells following exposure to SS (Fig 2). We performed RT-PCR and Western blotting to investigate the changes in expression of Becline-1 at both mRNA and protein levels, respectively, in cells following exposure to different doses of SS for 24 h (Fig 2A). Quantitative analysis of our RT-PCR and Western blotting data indicated that SS dose-dependently increased expression of Becline-1 at mRNA and protein levels (Fig 2B), confirming the onset of the functional autophagic activity in the cells. On the basis of our search in the miR database (http://mirdb.org), it is worth mentioning here that an overexpression of miR-30e can target and degrade Beclin-1 mRNA (Fig 2C), leading to inhibition of the functional autophagy in cancer cells.

### Effects of miR-30e transfection and PAC treatment on cell viability

**Fig 3:**
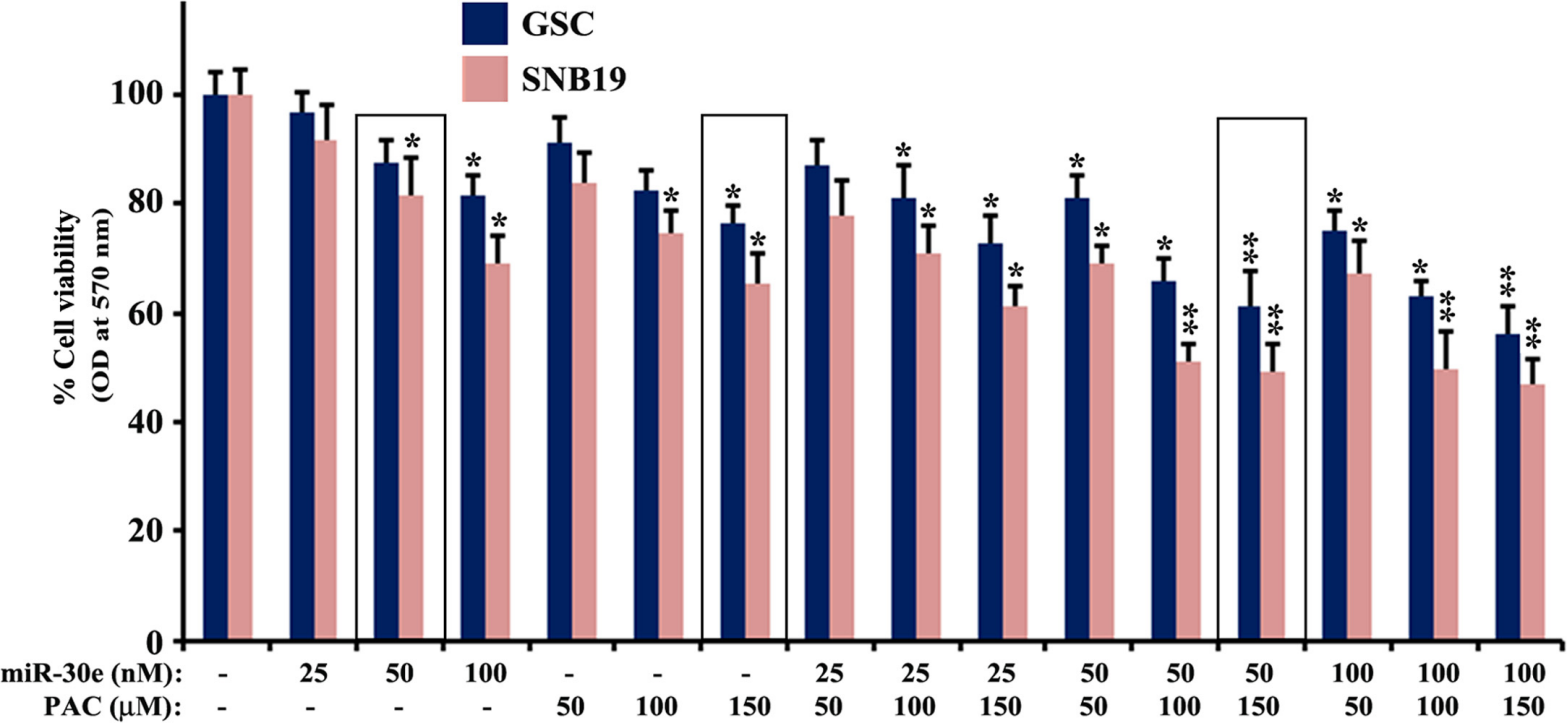
MTT assay to determine the changes in residual cell viability in the 2 mM SS pre-treated GSC and SNB19 cells after transfection with 25, 50, and 100 nM miR-30e for 12 h or treatment with 50, 100, and 150 μM PAC for 24 h, or their combinations. After the treatments and incubation, alterations in cell viability were measured by the MTT assay. All experiments were conducted in triplicates and the results were analyzed for statistical significance. Difference between control (CTL, the untreated group) and a monotherapy or combination therapy was considered significant at *P < 0.05 or **P < 0.01.

We performed cell viability studies following miR-30e (25, 50, and 100 nM) transfection and PAC (50, 100, and 150 μM) treatment alone and also in combination of both agents in the SS (2 mM) treated GSC and SNB19 cells (Fig 3). We did not notice any difference in cell viability between the SS untreated cells (CTL) and the SS treated cells. Based on this observation, we contemplated that SS treatment did not induce apoptosis and probably did resist induction of apoptosis. However, dose-dependently monotherapy (miR-30e or PAC) and also combination therapy (miR-30e and PAC) decreased cell viability in the SS pre-treated GSC and SNB19 cells (Fig 3). We analyzed the changes in cell viability and determined the combination index (CI) values of two therapeutic agents at different concentrations in both cell lines (Table 1). Based on the synergistic (CI < 1) values, combination of 50 nM miR-30e and 150 μM PAC exhibited the most synergistic effect in reducing cell viability in GSC and SNB19 cells. Therefore, we selected 50 nM miR-30e and 150 μM PAC alone and in combination in all subsequent experiments in both cell lines.

**Table 1:** Combination index (CI) of miR-30e and PAC in GSC and SNB19 Cells.

GSC			SNB19		
miR-30e (nM)	PAC (μM)	CI values	miR-30e (nM)	PAC (μM)	CI values
25	50	1.231	25	50	0.914
25	100	1.212	25	100	1.136
25	150	0.997	25	150	0.934
50	50	0.942	50	50	0.862
50	100	0.872	50	100	0.751
**50**	**150**	**0.814**	**50**	**150**	**0.653**
100	50	1.013	100	50	0.956
100	100	0.951	100	100	0.971
100	150	1.105	100	150	1.125

The CI values for GSC and SNB19 cells after treatment with combination of different doses of miR-30e (nM) and PAC (μM) (25 + 50, 25 + 100, 25 + 150, 50 + 50, 50 + 100, 50 + 150, 100 +50, 100 + 100, and 100 + 150). Conventionally, CI > 1 indicates antagonism, CI = 1 indicates additive effect, and CI < 1 indicates synergism. The lowest CI values (shown in **bold**) indicate the highest synergistic effects of combination of two agents.

### Combination of miR-30e and PAC inhibited SS-induced autophagy

**Fig 4:**
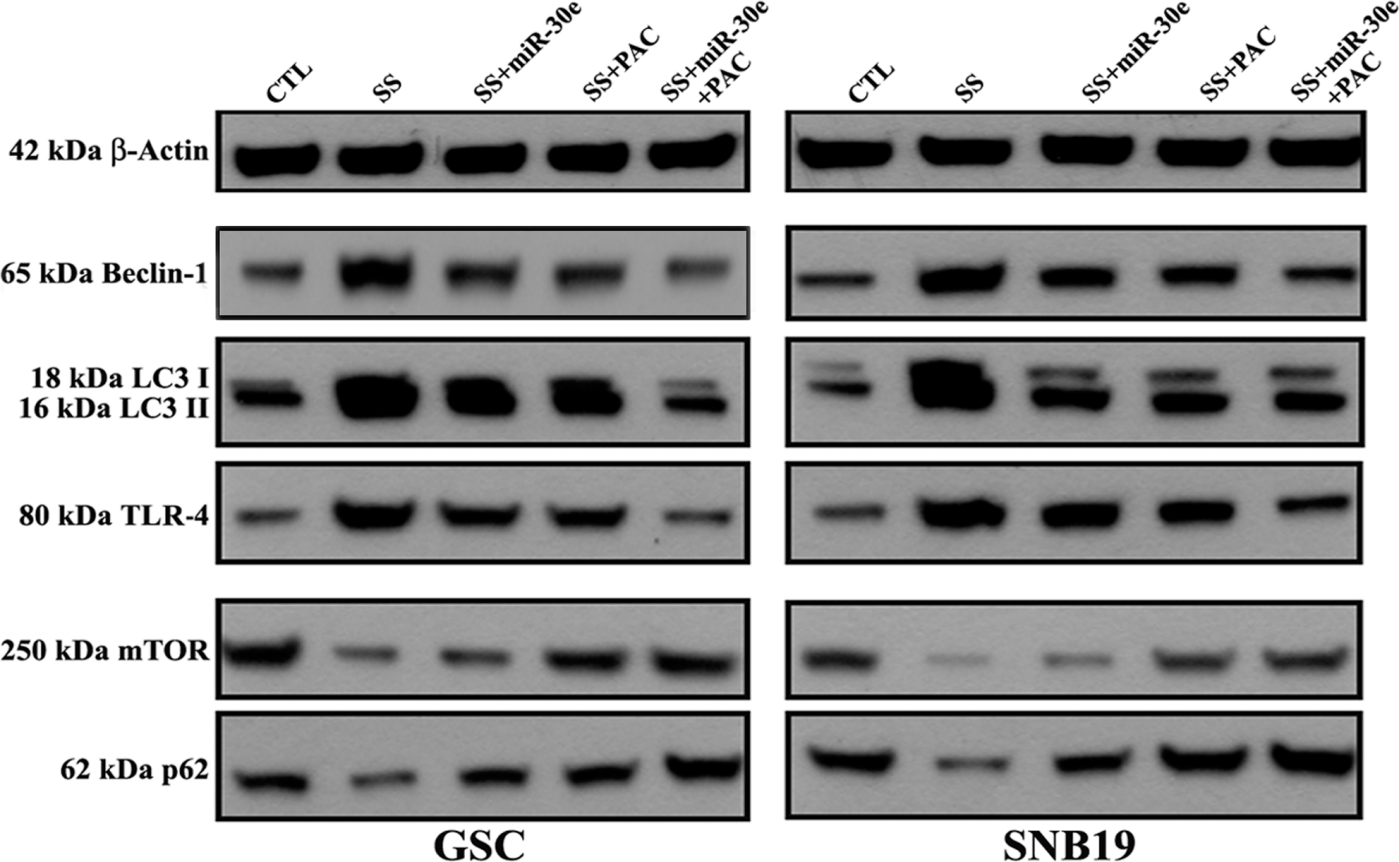
Western blotting to show changes in expression of key molecules in autophagic signaling pathway in GSC and SNB19 cells. Cells were treated as follows: untreated control (CTL); 2 mM SS for 24 h; 2 mM SS for 24 h + 50 nM miR-30e for 12 h; 2 mM SS for 24 h + 150 μM PAC for 24 h; and 2 mM SS for 24 h + 50 nM miR-30e for 12 h + 150 μM PAC for 24 h. Representative Western blots showed changes in expression of β-actin, Beclin-1, LC3 I and LC3 II, TLR-4, mTOR, and p62.

Next, we wondered whether or not the SS-induced autophagy could be inhibited after miR-30e transfection and PAC treatment alone and in combination in human GSC and SNB19 cell lines (Fig 4). We performed Western blotting to explore the specific molecular events that could be accountable for the inhibition of the SS-induced autophagy in both cell lines after miR-30e transfection and PAC treatment (Fig 4). Cells pre-treated with 2 mM SS for 24 h were subsequently transfected with 50 nM miR-30e for 12 h and treated with 150 μM PAC for another 24 h alone or in combination of both agents and then protein samples were used to perform Western blotting (Fig 4). Our Western blotting revealed that combination of miR-30e transfection and PAC treatment suppressed the autophagy inducing protein Beclin-1 in both cell lines, suggesting the inhibition of autophagy. During autophagy, LC3 is converted to LC3 I post-translationally, which is processed into the autophagosome membrane bound form LC3 II. Our Western blotting also showed that combination of miR-30e transfection and PAC treatment clearly inhibited the expression of LC3 II, implying that this combination therapy was highly effective in suppressing the SS-induced functional autophagic activity in both GSC and SNB19 cells. We also found significant inhibition of expression of toll-like receptor-4 (TLR-4) after combination therapy (Fig 4), indicating the blockage of a prominent molecule in autophagic signaling pathway. Finally, combination therapy increased the expression of mammalian target of rapamycin (mTOR), the negative regulator of functional autophagy, in both cell lines. We also examined the potential of the combination therapy on accumulation of p62 protein, also called sequestosome 1 or SQSTM1 (a well-known substrate in autophagy and also a molecular marker to study autophagic flux), and observed an increase in p62 accumulation, indicating inhibition of autophagy by the combination therapy. These results clearly indicated that the SS-induced functional autophagy was indeed inhibited after miR-30e transfection and PAC treatment in GSC and SNB19 cells.

### Combination of miR-30e transfection and PAC treatment induced apoptosis in SS pre-treated cells

**Fig 5:**
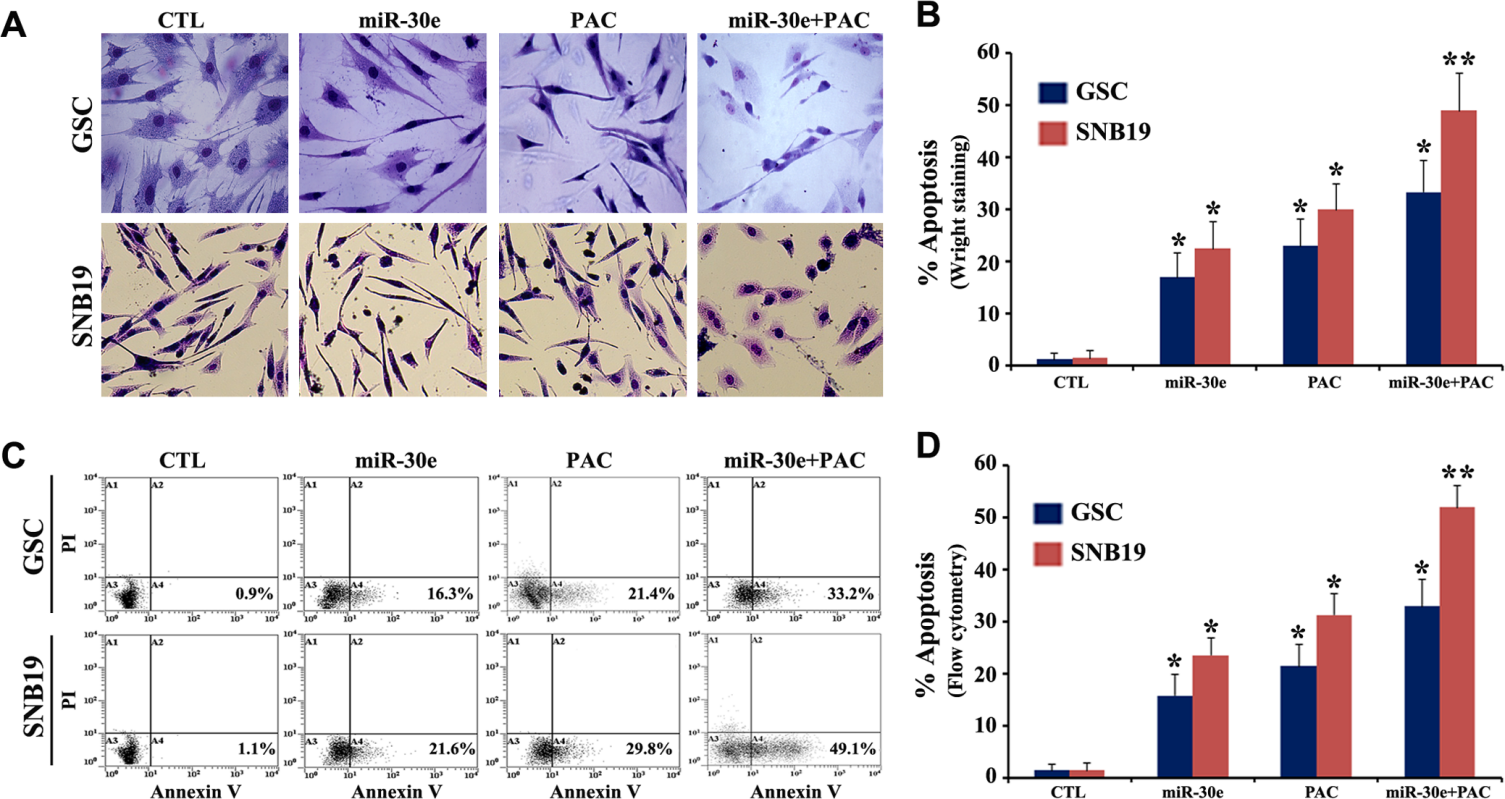
Detection and determination of apoptosis after overexpression of miR30e or/and treatment with PAC in the SS pre-treated GSC and SNB19 cells. Cells were treated as follows: untreated control (CTL); 2 mM SS for 24 h + 50 nM miR-30e for 12 h; 2 mM SS for 24 h + 150 μM PAC for 24 h; and 2 mM SS for 24 h + 50 nM miR-30e for 12 h + 150 μM PAC for 24 h. (A) *In situ* Wright staining was performed to show morphological features of apoptosis in both cell lines. (B) Determination of amounts of apoptosis based on *in situ* Wright staining. (C) Annexin V-FITC/PI binding assay followed by flow cytometry to show accumulation of apoptotic populations (A4). (D) Determination of amounts of apoptosis based on flow cytometry. All experiments were performed in triplicates. Significant difference between CTL and a treatment was indicated by *P < 0.05 or **P < 0.01.

After successfully inhibiting functional autophagy in the SS pre-treated cells, we anticipated that reduction in cell viability after miR-30e transfection and PAC treatment alone or in combination would be due to induction of apoptosis in both GSC and SNB19 cells. To assess this hypothesis that inhibition of autophagy could trigger induction of apoptosis, we performed *in situ* Wright staining and Annexin V-FITC/PI binding studies in the SS pre-treated cells after miR-30e transfection and PAC treatment (Fig 5). Our *in situ* Wright staining confirmed the morphological features of apoptosis such as cell shrinkage, chromatin condensation, and membrane blebbing in the cells following monotherapy or combination therapy (Fig 5A). Further, Annexin V-FITC/PI binding (a biochemical assay for apoptosis) followed by flow cytometric analysis showed varying number of cells accumulating in A4 quadrant of the flow cytometric dot-plot, indicating amounts of apoptosis due to miR-30e transfection or/and PAC treatment (Fig 5C). Our quantitative analysis of the *in situ* Wright staining and flow cytometric data indicated significant increase in apoptosis in both cell lines after combination therapy when we compared with monotherapy or CTL cells (Fig 5B and 5D). Interestingly, we observed that the combination therapy was more effective in SNB19 cells than GSC cells. Obviously, GSC cells are more resistant to induction of apoptosis. As there was no change in cell viability between the SS untreated cells and the SS treated cells, we thought that SS did not play a role in inducing apoptosis. So, all subsequent studies for induction of apoptosis were performed with the SS untreated cells as control (CTL).

### Combination of miR-30e transfection and PAC treatment decreased expression of the apoptosis inhibitors AVEN and BIRC6

**Fig 6:**
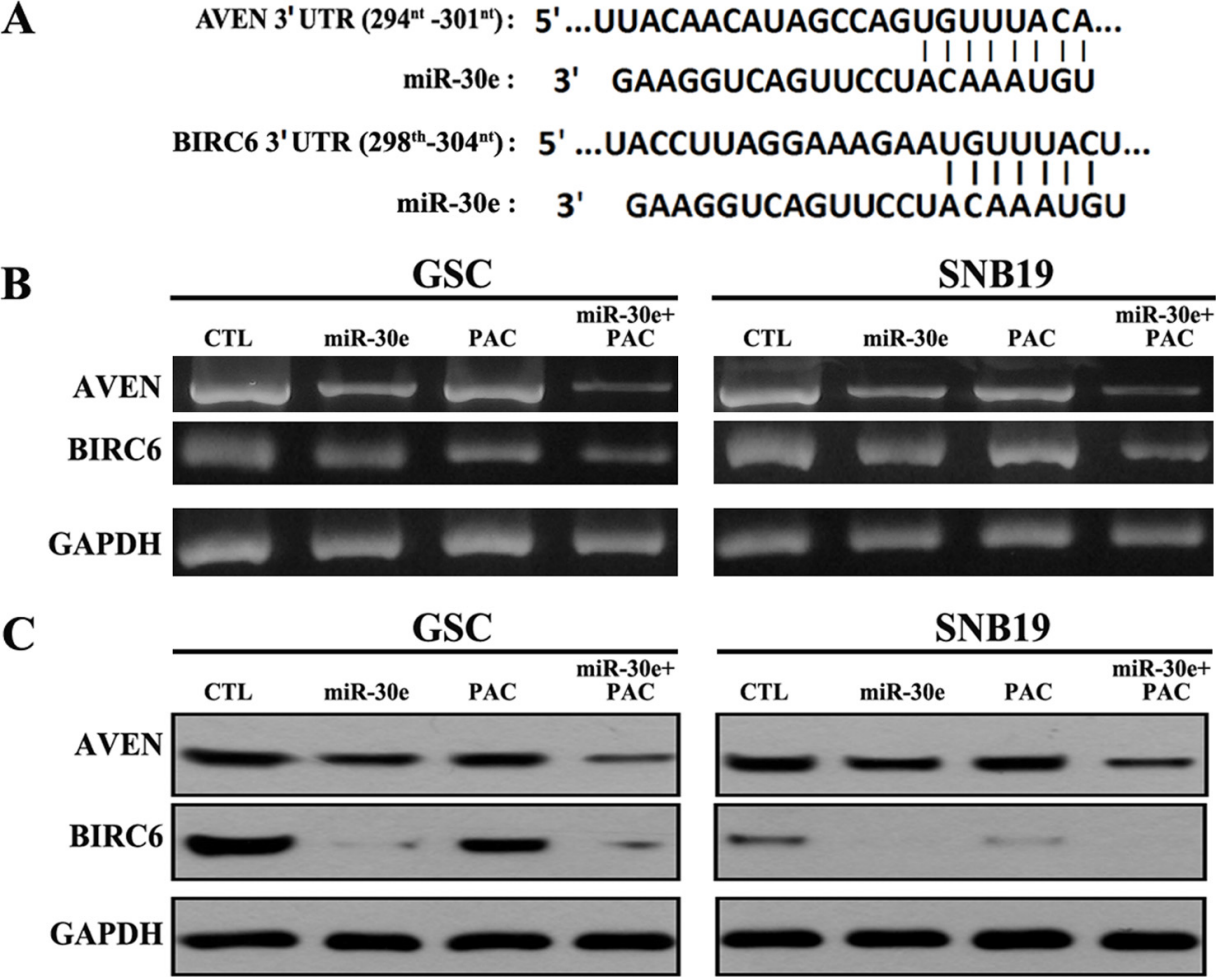
Target prediction for miR-30e and also RT-PCR and Western blotting to examine levels of expression of AVEN and BIRC6 in GSC and SNB19 cells. (A) Bioinformatics analysis showing AVEN and BIRC6 as potential targets for miR-30e. (B) RT-PCR analysis and band density quantification to determine relative mRNA expression of AVEN and BIRC6 in GSC and SNB19 cells. Treatments: untreated control (CTL); 2 mM SS for 24 h + 50 nM miR-30e for 12 h; 2 mM SS for 24 h + 150 μM PAC for 24 h; and 2 mM SS for 24 h + 50 nM miR-30e for 12 h + 150 μM PAC for 24 h. (C) Western blotting for examining protein expression of AVEN and BIRC6 in GSC and SNB19 cells. Treatments: untreated control (CTL); 2 mM SS for 24 h + 50 nM miR-30e for 12 h; 2 mM SS for 24 h + 150 μM PAC for 24 h; and 2 mM SS for 24 h + 50 nM miR-30e for 12 h + 150 μM PAC for 24 h. Western blots to show inhibition of protein expression of AVEN and BIRC6.

Next, we performed RT-PCR and Western blotting to explore alterations in expression of AVEN (apoptosis and caspase activation inhibitor) and BIRC6 (a member of the inhibitor-of-apoptosis proteins) at mRNA and protein levels, respectively, following miR-30e transfection and PAC treatment in the 2 mM SS pre-treated GSC and SNB19 cells (Fig 6). Many earlier reports suggested that miR-30 family could be a potent tumor suppressor in some cancers and its direct overexpression could induce the molecular mechanisms of extrinsic and intrinsic pathways of apoptosis. We performed an extensive bioinformatics analysis (http://mirdb.org) and observed that the apoptosis inhibitors AVEN and BIRC6 could be potential targets of miR-30e (Fig 6A). Indeed, our results from RT-PCR and Western blotting clearly indicated dramatic degradation of both AVEN and BIRC6 following miR-30e transfection in both cell lines. Most significant suppression of these apoptosis inhibitors (AVEN and BIRC6) were observed after combination therapy with miR-30e and PAC. Thus, we suggested that miR-30e overexpression potentiated activity of PAC for increasing induction of apoptosis in GSC and SNB19 cells.

### miR-30e overexpression potentiated PAC for increasing induction of apoptosis by modulating both extrinsic and intrinsic pathways of apoptosis

**Fig 7:**
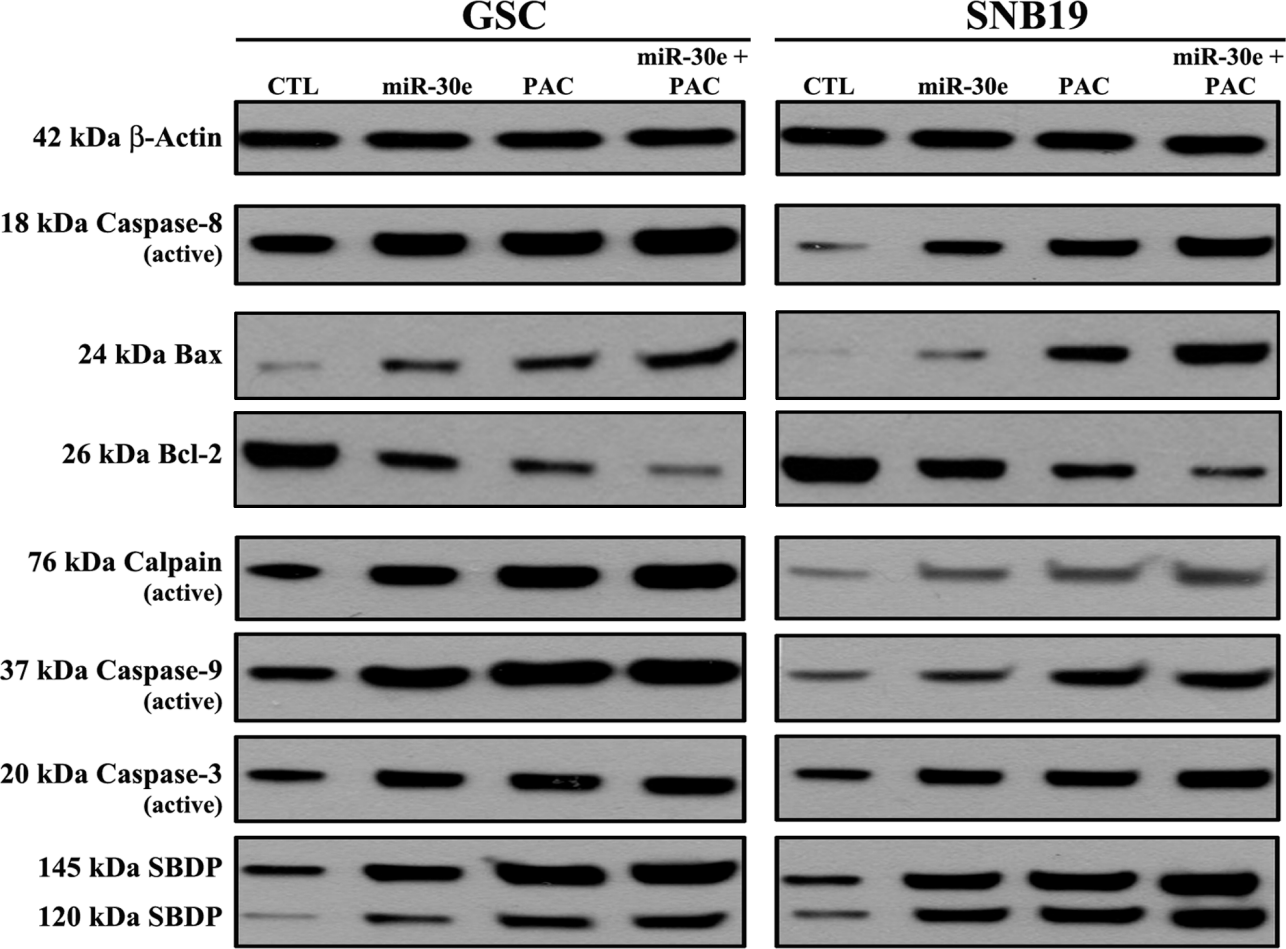
Changes in levels of the molecular components of apoptosis following transfection with miR-30e or/and treatment with PAC in the SS pre-treated GSC and SNB19 cells. Treatments: untreated control (CTL); 2 mM SS for 24 h + 50 nM miR-30e for 12 h; 2 mM SS for 24 h + 150 μM PAC for 24 h; and 2 mM SS for 24 h + 50 nM miR-30e for 12 h + 150 μM PAC for 24 h. Western blots to show changes in molecules involved in apoptosis.

To determine the plausible molecular mechanisms of induction of apoptosis in the SS pre-treated GSC and SNB19 cells following miR-30e transfection or/and PAC treatment, we performed Western blotting using the primary antibodies against the key molecules involved in regulation of apoptotic death (Fig 7). Our results showed activation of extrinsic pathway of apoptosis with generation of active caspase-8 fragment in varying degrees in both cells following monotherapy and combination therapy (Fig 7). Activation of intrinsic pathway of apoptosis also occurred as evidenced from an increase in expression of Bax (pro-apoptotic protein) and decrease in expression of Bcl-2 (anti-apoptotic protein) in both cell lines (Fig 7). Combination of miR-30e transfection and PAC treatment caused the highest activation of calpain, caspase-9, and also caspse-3 (the final executioner of apoptosis). We found that activation of both calpain and caspase-3 caused proteolysis of the cytoskeletal protein α-spectrin at specific sites to generate the calpain-specific 145 kDa spectrin break down product (SBDP) and the caspase-3-specific 120 kDa SBDP, respectively, in both cell lines for induction of apoptosis. Almost uniform expression of β-actin served as an internal control and indicated equal protein loadings.

## Discussion

In the present study, we employed the chemotherapy and radiotherapy resistant human GSC and also glioblastoma SNB19 (PTEN mutant) cells to evaluate the therapeutic efficacy of the combination of miR-30e (a tumor suppressor miR) and PAC (an apoptosis inducing plant-derived polyphenolic compound). Our study demonstrated that combination of miR-30e and PAC acted synergistically for inhibition of functional autophagy and induction of apoptosis to control growth of both GSC and SNB19 cells in hypoxia condition. This combination therapy very effectively inhibited autophagy by suppression of Beclin-1 and induced apoptosis by inhibition of AVEN and BIRC6 and upregulation of caspase activity. We observed that GSC cells were more resistant to the anti-tumor effects of monotherapy or combination therapy. Ectopic overexpression of miR-30e potentiated PAC to block an important cell survival strategy (autophagy) and promoted a highly desirable cell death process (apoptosis) in both human GSC and glioblastoma SNB19 cells.

Hypoxia-induced autophagy is a survival mechanism that promotes tumor progression [23]. Many investigators used the hypoxia-mimicking chemical agent such as CoCl_2_ to induce hypoxic stress in cell culture models [24–26]. Such chemical hypoxia, however, may result in heavy metal toxicity or impose health security threats. Use of sodium sulfite (SS) as an alternate offers a more suitable model for inducing hypoxia [27]. This study demonstrated that the SS-induced hypoxia was associated with the induction of hypoxia-inducible factor-1 alpha (HIF-α) [27]. Induction of HIF-α is well-known to induce autophagy [24]. So, we generated hypoxia-induced autophagy using SS in our GSC and SNB19 cells and also we could adjust the degree of hypoxia by using the appropriate concentration of soluble SS for induction of autophagy. We observed that use of 2 mM SS could induce 16% and 24% autophagy in GSC and SNB19 cells, respectively. It should be noted here that we detected and determined autophagic cells containing autophagosomes with the use of AO staining, which could stain not only autophagosomes but also other acidic vesicles (e.g., lysosomes, endosomes). It is recommended that Cyto-ID autophagy detection kit (Enzo) should be used for selective labeling and detecting autophagic vacuoles while Lyso-ID green detection kit (Enzo) should be used for selective staining of acidic organelles (e.g., lysosomes, endosomes) in live cells to access a clear picture of functional autophagy. Besides, examination of some important molecular markers such as Beclin-1, LC3 II, and LAMP1 can support functional autophagy. Beclin-1 plays a crucial role in early phase in induction of autophagy [28]. Our results showed upregulation of Beclin-1 at mRNA and protein levels in the GSC and SNB19 cells following exposure to SS, confirming presence of functional autophagy.

A few earlier studies reported an important role of the miR-30 family in sensitizing tumor cells to the conventional therapeutic agents via suppression of Beclin-1 mediated autophagy [29,30]. Our search of the miR database (**http://mirdb.org**) indicated that miR-30e could inhibit expression of Beclin-1 (autophagy promoter) and also AVEN and BIRC6 (apoptosis inhibitors) by their message (mRNA) cleavage or translational (protein) repression. Therefore, we explored the possibility of ectopic overexpression of miR-30e to suppress autophagy (a potential cell survival strategy) and promote apoptosis (a desirable cell death process) through modulation of expression of Beclin-1, AVEN, and BIRC6 in human GSC and SNB19 cells. Although many investigators reported a significant anti-cancer potential of PAC isolated from grape seeds, only a few studies were performed in human glioblastoma cells [21,22]. We observed that combination of 50 nM miR-30e and 150 μM PAC acted synergistically and most significantly inhibited cell growth in GSC and SNB19 cells where CI values were 0.81 and 0.65, respectively. Beclin-1 participates in the early stages of autophagy, promoting the nucleation of the autophagic vesicle and recruiting proteins from the cytosol [31]. Thus, an effective inhibition of Beclin-1 by combination of miR-30e and PAC could inhibit early stages of autophagy. Also, the inhibition of an upstream molecule (Beclin-1) is supposed to be associated with the blockage of downstream molecules (e.g, LC3 II) in autophagy pathway. LC3 II is considered as a marker of final autophagosome formation and it is also inhibited by our combination of miR-30e and PAC in GSC and SNB19 cells. TLRs are expressed in colon cancer, breast cancer, prostate cancer, melanoma, and lung cancer [32]. We found that suppression of TLR-4 inhibited autophagy in GSC and SNB19 cells following combination therapy with miR-30e and PAC. Our results from Western blotting also showed upregulation of autophagy suppressive markers such as mTOR and p62 after miR-30e transfection and PAC treatment in both GSC and SNB19 cells.

We also observed that combination of miR-30e transfection and PAC treatment very effectively increased apoptosis in the SS pre-treated GSC and SNB19 cells. *In situ* Wright staining indicated the presence of significantly high number of cells exhibiting morphological features of apoptosis after the combination therapy. Annexin V-FITC/PI binding assay, which was used to detect an early biochemical evidence of apoptosis, also confirmed the induction of apoptosis in both GSC and SNB19 cells. These results are in complete harmony with the previous reports where inhibition of autophagy has been linked to a trigger for induction of apoptosis so as to prevent the growth of tumor cells [32–34].

A recent study reported that very low level of miR-30e was associated with resistance to apoptosis and enforced expression of miR-30e induced apoptosis in cancer cells [35]. Our data showing induction of apoptosis in GSC and SNB19 cells after enforced expression of miR-30e suggested that miR-30e levels were very low or negligible in these glioblastoma cells. We found that efficacy of miR-30e overexpression and PAC treatment was correlated well with the expression of key apoptotic signaling molecules involved in the extrinsic (death receptor mediated) and intrinsic (mitochondria mediated) pathways of apoptosis. Increases in expression of active caspase-8 following the combination therapy indicated the activation of the death receptor mediated pathway of apoptosis. Our data also suggested that combination therapy could upregulate Bax (pro-apoptotic protein) and down regulate Bcl-2 (anti-apoptotic protein) resulting in an increase in Bax:Bcl-2 ratio to trigger the release of mitochondrial pro-apoptotic factors. Another significant result from our study was the activation of calpain, a cysteine protease known to play an important role in induction of apoptosis [36–38], for induction of apoptosis in GSC and SNB19 cells after combination therapy with miR-30e and PAC. Our data thereby showed that this combination therapy induced the mitochondria mediated pathway of apoptosis for activation of caspase-9, which in turn activated caspase-3 for completion of apoptotic process in GSC and SNB19 cells. Combination of miR-30e transfection and PAC treatment enhanced proteolytic activities of calpain and caspase-3 for cleavage of α-spectrin at specific sites to generate calpain-specific 145 kDa SBDP and caspase-3-specific 120 kDa SBDP, respectively, to induce apoptotic death in both GSC and SNB19 cells.

## Conclusions

**Fig 8:**
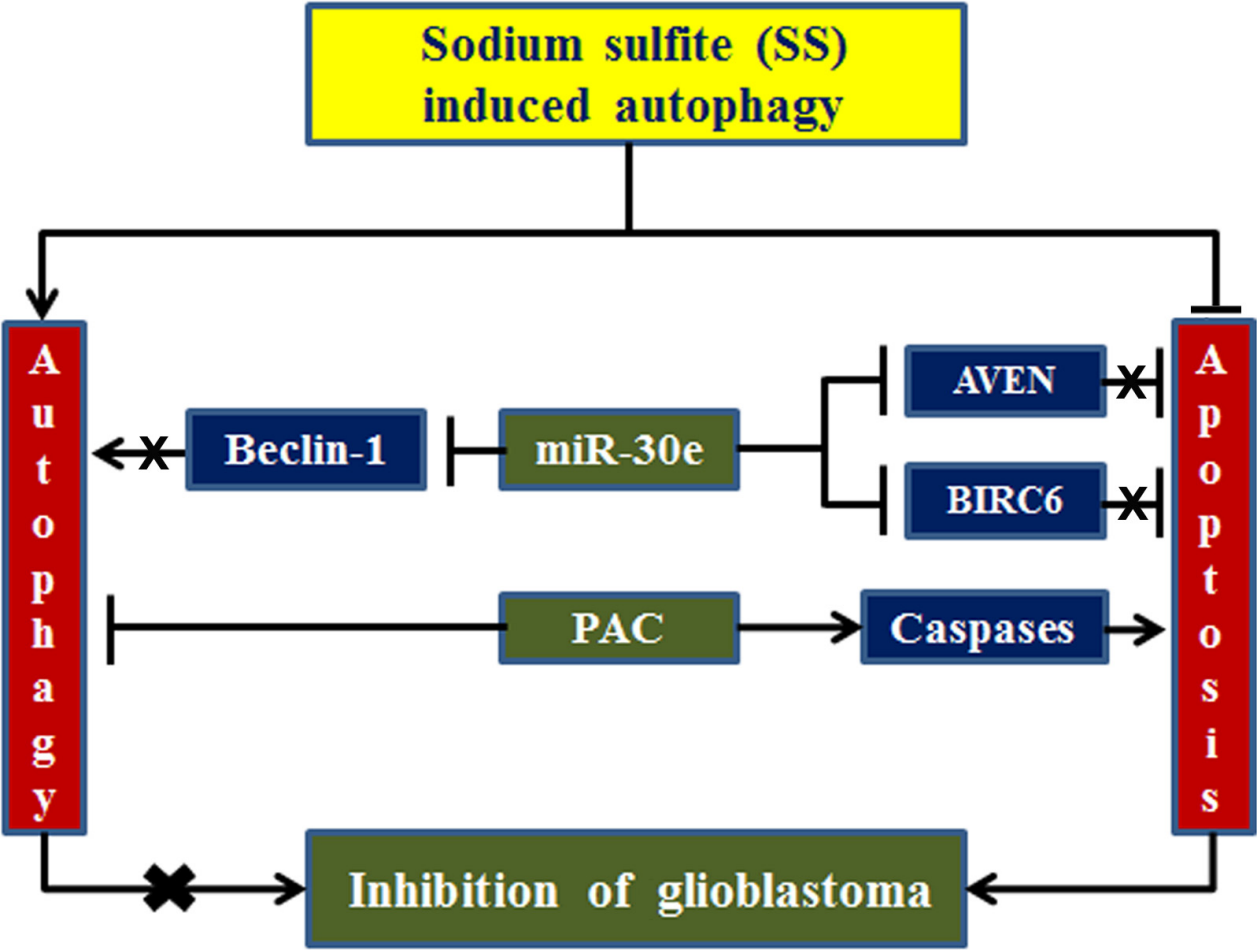
Schematic presentation to show inhibition of SS-induced autophagy and induction of apoptosis in glioblastoma cells. miR-30e inhibited autophagy via degradation of Beclin-1 and induced apoptosis via degradation of AVEN and BIRC6. PAC exerted its anti-cancer activity through activation of caspases. Combination of miR-30e and PAC most effectively inhibited autophagy and augmented apoptosis for controlling growth of glioblastoma cells.

In summary, combination therapy with miR-30e overexpression and PAC treatment could block autophagic cell survival strategy and induce apoptotic cell death process in human GSC and SNB19 cells under hypoxia condition (Fig 8). Our current results clearly indicate that SS can be used to mimic hypoxia-induced autophagy in both GSC and SNB19 cells. Induction of autophagy is a potential survival strategy in glioblastoma harboring heterogeneous cells. Thus, inhibition of autophagy threatens growth of glioblastoma. Combination of miR-30e transfection and PAC treatment can completely inhibit SS-induced autophagy and increase induction of apoptosis for controlling the growth of human glioblastoma cells. So, our current study strongly suggests that combination of miR-30e transfection and PAC treatment can be further explored as a novel therapeutic strategy for treatment of glioblastoma in humans in the near future.

## Abbreviations

AO: acridine orange
AVEN: apoptosis and caspase activation inhibitor
AVO: acidic vesicular organelles
BIRC6: baculoviral inhibitor-of-apoptosis repeat-containing protein 6
CoCl_2_: cobalt(II) chloride
DMEM: Dulbecco's modified eagle medium
DMSO: dimethyl sulfoxide
FITC: fluorescein isothiocyanate
GSC: glioblastoma stem cells
HIF-α: hypoxia-inducible factor-1 alpha
HRP: horseradish peroxidase
LC3: microtubule associated protein 1 light chain 3
miRDB: microRNA database
mTOR: mammalian target of rapamycin
MTT: 3-(4,5-dimethylthiazolyl-2)-2,5-diphenyl-tetrazoliumbromide
p62: protein (also called sequestosome 1 or SQSTM1) with a molecular weight of 62 kDa
PAC: proanthocyanidin
PI: propidium iodide
PI3K: phosphoinositide 3-kinase
RT-PCR: reverse transcription-polymerase chain reaction
SBDP: spectrin breakdown product
SS: sodium sulfite
TLR-4: toll-like receptor-4
